# Awareness, perception and attitude towards nano-dentistry among post-graduates from Rajasthan, India

**DOI:** 10.6026/9732063002001537

**Published:** 2024-11-30

**Authors:** Saloni Kanodia, Abhishek Khairwa, Soumyaranjan Nanda, Shubhangi Pareek, Marium Mishra, Shreeyam Mohapatra, Pratik Surana

**Affiliations:** 1 Department of Pediatric and Preventive Dentistry, Jaipur Dental College, Maharaj Vinayak Global University, Dhand amer, Jaipur, Rajasthan-302028, India; 2 Department of Conservative Dentistry and Endodontics, SCB Dental College and Hospital, Cuttack, Odisha, India; 3 Department of Oral Pathology & Microbiology, SCB Dental College & Hospital, Cuttack, Odisha; 4 Kalinga Institute of Dental Sciences, KIIT Deemed to be University, Bhubaneswar, Odisha, India; 5 Department of Oral Medicine and Radiology, SCB Dental College & Hospital, Cuttack, Odisha, India; 6 Department of Pedodontics and Preventive Dentistry, Maitri College of Dentistry and Research Centre, Durg, Chhattisgarh, India

**Keywords:** Nano-dentistry, nano-robotics, postgraduates

## Abstract

Nano-dentistry utilizes nanoparticles and nano-materials to revolutionize dental treatments, offering enhanced precision, durability and effectiveness in oral health care solutions. A survey of 290 postgraduate dental students in Rajasthan revealed that 93.4% were knowledgeable about nano-dentistry, with over 80% expressing positive views on its applications in eight key areas. These findings indicate a strong awareness and positive perception towards nanotechnology in dental care. The study highlights the growing interest and underscores the need to integrate nanotechnology into dental curricula to maximize its benefits for oral health care.

## Background:

The term "nano" originates from the Greek word meaning "dwarf". The prefix "nano" denotes a factor of ten to the negative ninth power, or one billionth and is typically combined with a noun to create words such as nanometer, nanotechnology and nano-robot [1]. The concept of 'nanotechnology' was introduced by Professor K. Eric Drexler. Additionally, Robert A. Freitas defines nano-dentistry as the science and technology enabling comprehensive oral health maintenance through the use of nano-materials, biotechnology, including tissue engineering and eventually dental nano-robots [1- 2]. A new field called nano-medicine, which also includes nano-dentistry, uses biotechnology, genetic engineering, nano-scale-structured materials and ultimately sophisticated molecular machines and nano-robots to detect, cure and prevent illness and trauma, as well as to reduce pain and maintain and improve human health [3]. Nanotechnology is a new arena that has backed in the development of new remedial and individual agents, with the added benefit of perfecting medicine accretion when minimizing the negative goods that small - patch specifics have. The enclosed motes ' bitsy size, increased chemical stability and apparent solubility, along with the multi-functionality of nanoparticles, are features that open up new perspectives for natural exploration. Nanoparticles have a mainly advanced face area per unit mass than larger patches owing to their small size. Likewise, in the nano-scale, amount goods come more prominent. Nanotechnology in dentistry has several downsides, including high product costs and a lack of understanding regarding the effectiveness. Nanotechnology and nano-dentistry are hardly mentioned in the class at colorful dental academy situations [2]. Nanotechnology is a novel arena that has aided in the development of new therapeutic and diagnostic agents, with the added benefit of improving drug accretion when minimizing the negative effects that small-molecule medications have. The enclosed molecules' tiny size, increased chemical stability and apparent solubility, along with the multi-functionality of nanoparticles, are features that open up new perspectives for biological research [2-3]. Therefore, it is of interest to assess the awareness, perception and attitude towards nano dentistry among postgraduate dental students in India based on questionnaire.

## Materials and Methods:

Structured questionnaire comprising of 20 questions was validated by subject experts in the field of dentistry. Postgraduate students in the field of dentistry in Rajasthan were approached and provided with the validated questionnaire for an online survey through Google form portal. The total number of students participated in the survey is 300. Further, descriptive and inferential statistical analyses were carried out.

## Results:

From 300 postgraduate dental students, 299 participated in the study. One postgraduate dental student was excluded from the study as he had not given consent to fill the forms and incomplete forms submitted. Most correctly answered question was question no 5 and most incorrectly answered question was question no 14 (Table 1).

## Discussion:

Dentistry has advanced in terms of technology over time, making it more accessible. Dentistry has transitioned through several years. It is experiencing yet another transformation in terms of assisting humanity, this time using nanotechnology in conjunction with nano-materials, biotechnology and nano-robotics [4]. The concept of nanotechnology was first introduced by Richard Feynman in 1959 during a lecture at the California Institute of Technology, where he expounded on the implications of manipulating information at a diminutive scale and the impending utilization of minuscule robots and computers. Since then, this field has witnessed substantial progress and now pervades all aspects of technological development. In contemporary dentistry, innovative treatment approaches facilitated by nanotechnology encompass localized anesthesia, natural restoration of dentition, permanent resolution of hypersensitivity, complete orthodontic realignment within a single session, application of covalently bonded diamond-coated enamel and continuous oral health maintenance through the deployment of mechanical dentifrobots (nano-robotic dentifrice), which eradicate caries-causing bacteria and repair areas affected by decay [5].

Present survey showed 83.6% (250) students have knowledge of its advantage. 255 (85.3%) students know advantages of nano adhesives. Nanoparticles in dentistry: Particles ranging from 0.1 nm to 100 nm in size - 197(65.9%) students responded correctly to this (nanoparticles < 100 nm particle size).279(93.3%) students responded that Nanotechnology has its applications in both medicine and dentistry. The advancement of nano-dentistry, through the utilization of nano-materials and biotechnologies such as tissue engineering and nano-robots, promises to significantly enhance oral health. Concurrently, innovations in biomaterials and biotechnology have given rise to the emergent discipline of nano-medicine. A survey indicates that 207 students (69.2%) concur that dentifrices incorporating nano-robots can effectively mitigate halitosis by deploying intelligent nano-scale devices to target and eliminate pathogenic bacteria, thereby promoting the growth of benign oral microflora. Main obstacle to implement nanotechnology in dental practice is allergy due to some nano-materials and lack of awareness-259 (86.6%) students agreed to this in our study. 263 (88%) participants knew the ways to increase knowledge of nanotechnology in dentistry through journals, conferences and CDE programs, by inclusion in academic curriculum in dental schools, advertisements and public notice. 233(77.9%) students correctly answered that high resolution Electron microscope is required to visualise nanoparticles. Presently, nanotechnology has ushered in a new age for TE/RM by enabling the creation of structures that are the same size as naturally occurring tissues. It is possible to create nano-scaffolds that closely resemble tissue-specific extracellular matrix. The smaller size of the nanoparticles enables them to react quickly to environmental stimuli such as pH, magnetic fields, ultrasounds and exposure to X-rays. Drugs, genetic material or biological factors can be delivered systemically or locally in a regulated manner using nano-scaffold materials [6]. By encapsulating or attaching to surfaces, nano-scaffolds can stabilize bioactive chemicals, target their transport from cells, encourage internalization of molecules and regulate the release of biological factors at the desired location [7]. Nano adhesives offers several advantages over conventional dental adhesives it has properties like improved bond strength, enhaced durability, better marginal durability increased biocompatibility. Nano-adhesives are utilized in various dental procedures, including: Direct Restorations: Used in bonding composite resins to the tooth structure, providing durable and aesthetically pleasing restorations for cavities and other defects. Indirect Restorations: Employed in cementing inlays, onlays, crowns and veneers, ensuring a strong and reliable bond between the restoration and the tooth. Orthodontics: Applied in bonding orthodontic brackets to teeth, enhancing bond strength and reducing bracket failure rates. Endodontics: Used in sealing root canal fillings, improving the seal and reducing the risk of microleakage and infection [8].

Nano-needles, which are nano-scale stainless steel needles, hold the potential to enable cellular surgery in the foreseeable future. These nano-needles are commercially available under the brand name Sandvik Bioline, specifically RK 91TM needles (AB Sandvik, Sandviken, Sweden). They are capable of delivering molecules, such as nucleic acids, proteins, or other chemicals, directly to the nucleus and can also be utilized for cellular surgical procedures. The primary advantage of the nano-needle methodology lies in its ability to target precise locations within the nucleus [9]. Additionally, nano-robotic dentifrice commonly referred to as dentifrobots, can be administered via mouthwash or toothpaste on a daily basis to maintain oral hygiene. These nano-robots are designed to protect both supragingival and subgingival surfaces by metabolizing targeted organic substances into benign and odorless vapors, while simultaneously performing continuous calculus debridement [10].

Nanotechnology-enhanced implants have the potential to significantly expedite bone growth and enhance predictability. The nano-scale modification of titanium endosseous implant surfaces can influence cellular and tissue responses, which may facilitate osseo-integration and improve dental implant therapy outcomes. Additionally, the incorporation of nanoscale deposits of hydroxyapatite (HA) and calcium phosphate creates a more intricate surface conducive to osteoblast formation [11].

## Challenges and considerations:

While the potential impacts of nano-dentistry are profound, several challenges must be addressed to fully realize its benefits. [12, 13-14].

[1] Safety and Biocompatibility: Ensuring the long-term safety and biocompatibility of nano-materials used in dental applications is crucial. Extensive research is required to understand the potential toxicological effects of these materials on human health. 

[2] Regulatory and Ethical Issues: The development and implementation of nano-dentistry must navigate complex regulatory and ethical landscapes. Establishing clear guidelines and standards for the use of nanotechnology in dental care is essential to ensure patient safety and public trust.

[3] Cost and Accessibility: The high cost of developing and producing nano-materials and nano-devices may limit their accessibility, especially in low-resource settings. Efforts to reduce costs and improve affordability are necessary to make nano-dentistry widely available.

## Conclusion:

Nano-dentistry has the potential to transform dental care through enhanced diagnostics, improved treatments, advanced preventive measures and regenerative therapies. While challenges remain, ongoing research and technological advancements are likely to overcome these barriers, leading to broader adoption and more significant impacts on oral health. From this survey we can conclude that: Dental postgraduates possess sufficient theoretical knowledge pertaining to nanotechnology; however, their understanding of its practical applications in Dentistry remains limited. There is an urgent need for increased clinical exposure to nano-materials within postgraduate clinics.

## Figures and Tables

**Figure 1:**
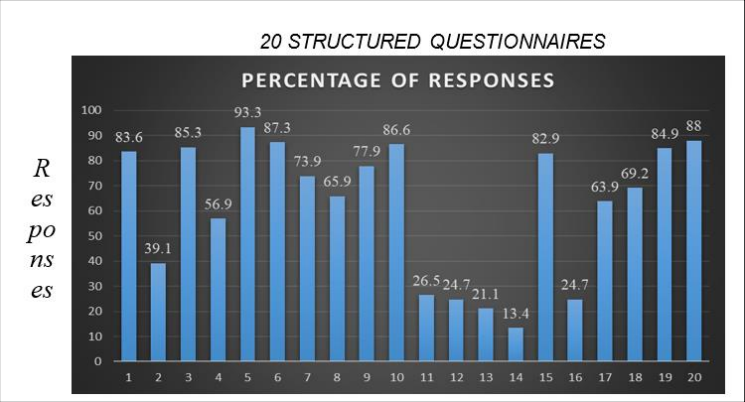
Represents percentage of post graduate students responded from various specialization branches. Highest response was from department of conservative dentistry and Endodontic while lowest responses were from department of public health dentistry.

**Figure 2:**
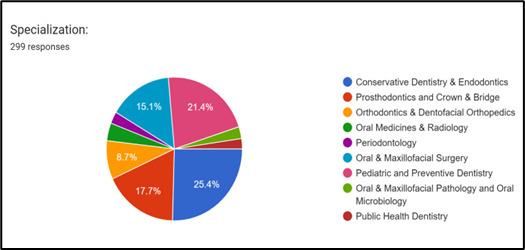
Represents percentage of responses to 20 structured questionnaires. Highest response was given to question number 5 whereas lowest response was noted with question no 14.

**Table 1:** Structured questionnaire with percentage of responses

Q1	**Nano-composite artificial teeth is superior to conventional acrylic teeth in terms of surface smoothness and abrasion resistance**	**250(83.6%)**
Q3	Advantages of nano adhesives	255(85.3%)
Q5	Nanotechnology have its application in dentistry and medicine	279(93.3%)
Q6	Properties of nanomaterial differ from other materials	261(87.3%)
Q10	Lack of awareness and allergy due to some of the nano-materials can be the main obstacle to implement nanotechnology in dental practice	259(86.6%)
Q15	Advantages of using nano- carbon tubes	248(82.9%)
Q19	Implants incorporating nanotechnology have the potential to facilitate accelerated bone growth, enhance predictability and decrease the duration required for osseointegration.	254(84.9%)
Q20	What are ways to increase knowledge of nanotechnology in dentistry	263(88%)
Q2	Where can nanomaterial-based tissue scaffold used	117(39.1%)
Q11	What is an OFNASET device	79(26.5%)
Q12	What are nano-needles	74(24.7%)
Q13	Are nanoparticles dangerous for human health	63(21.1%)
Q14	Which one, Carbon nanotubes, Nano-rods or Nano-bots used in drug delivery	40(13.4%)
Q16	Have you utilized nano materials in your clinical practice	74(24.7%)
